# The stromal fibrosis in aging ovary

**DOI:** 10.18632/aging.101370

**Published:** 2018-01-19

**Authors:** Takashi Umehara, JoAnne S. Richards, Masayuki Shimada

**Affiliations:** 1 Graduate School of Biosphere Science, Hiroshima University, Higashi-Hiroshima, Japan; 2 Department of Molecular & Cellular Biology, Baylor College of Medicine, Houston, TX77030, USA

**Keywords:** infertility, endocrinosis, oocyte, decreased ovarian reserve (DOR)

Female fertility declines with increasing age. It has been thought that the decline of female fertility is caused by the loss of follicles/oocytes in ovary; however, several reports indicate that about 10,000 follicles are present in ovary of women over 40 years of age [1]. In the women over 40 years of age, irregular menstrual cycles are related to abnormal endocrine profiles (high FSH/high LH/low AMH) [2]. Exogenous hormone treatment does not rescue the ovarian function in these women and predicts a low number of matured oocyte with poor quality in assisted reproductive technology (ART). In mice, around 10 months of age, the ovary contains a large number of small follicles. However, the female mice exhibit an extended estrous phase and a poor response to standard super-ovulatory regimens, suggesting that the decline in fertility of aged women and mice is related not only to the loss of follicles in ovary but also to the changes of ovarian function. In our previous studies [3], granulosa cell-specific *Nrg1* knockout (KO) mice (gc*Nrg1*KO) exhibited longer estrous cycles and a reduced number of pups born, with a linear decline from 6-10 months of age. To determine whether the mutant mice represent a model of ovarian aging, markers of ovarian aging were analyzed in ovaries of 6-month-old gc*Nrg1*KO mice. The mutant mice exhibit premature signs of ovarian aging that are observed in older WT mice and women, including a reduced response to exogenous hormonal treatment; elevated serum levels of LH, FSH, and testosterone; and reduced levels of inhibin B and AMH, that is a marker of the ovarian reserve. Therefore, we analyzed the mechanisms that contribute to reduced ovarian function with increasing age using gc*Nrg1*KO mice as a model of ovarian aging.

**Figure 1:**
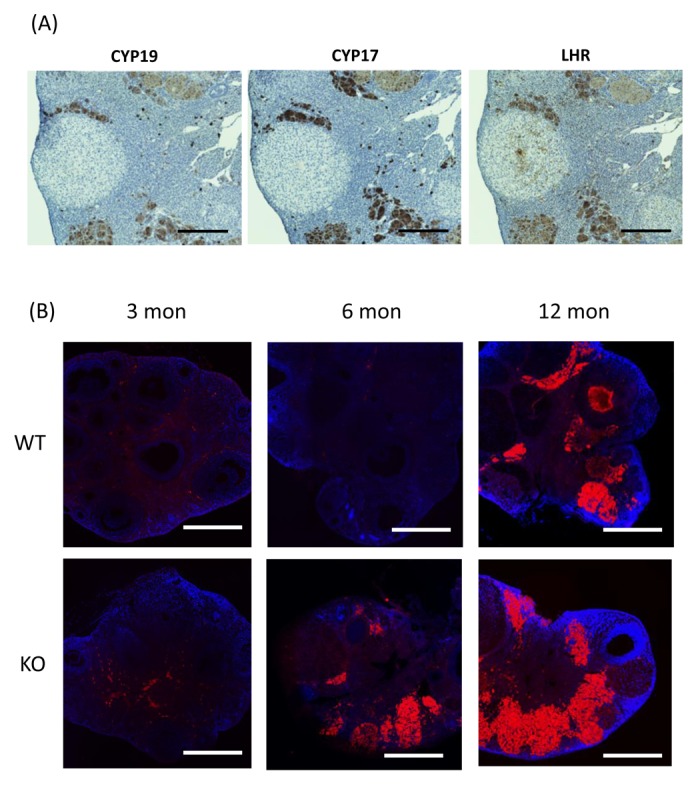
**The morphological changes in the ovary with increasing age.** (**A**) The localization of the heterogeneous, CYP19-positive, CYP17-positive, and LHR-positive cells in ovaries of 6-month-old gc*Nrg1*KO mice. Scale bars correspond to 300 µm (×100). (**B**) The images stained using rhodamine phalloidin, that is a marker of F-actin, and DAPI with constant settings that were established for the 12-month old gc*Nrg1*KO. Scale bars in image are 300 µm.

In the ovaries of 6-month-old gc*Nrg1*KO mice, follicular development was blocked in bilayer secondary follicles and heterogeneous cells accumulated in the ovarian stroma. Immunohistochemical analysis and picrosirius red staining, that visualizes collagen in paraffin-embedded tissue sections, showed that the heterogeneous cells in ovarian stroma were distinguished as two different types: LH receptor-positive endocrine cells (Figure 1A), and actin-rich fibrotic cells expressing collagen (Figure 1B). This ovarian phenotype was also observed in 12-month-old WT mice, suggesting that the endocrine and fertility changes that occur with increasing age are associated with the aberrant accumulation of endocrine cells in the ovarian stroma and their high expression of *Cyp17a1*, *Cyp19a1*, and *Lhcgr*. The accumulation of endocrine cells in the ovarian stroma has previously been observed in ovaries of LH beta (LHβ) over-expressing mice with increasing age [4]. Additionally, the fibrosis in the ovarian stroma and increased levels of basal LH are also observed in the patients with hypersecretion of LH [5]. Based on these considerations, we hypothesized that the accumulation and function of these cells appeared to be driven by elevated LH, and that the fibrosis suppressed follicular development. To analyze this hypothesis, 6-month-old gc*Nrg1*KO and 12-month-old WT mice were treated for 8-days with a GnRH-antagonist; ovarian histology showed that this regimen caused both the aberrant endocrine cells and fibrotic cells to disappear. Moreover, by 4 days after final GnRH treatment, follicular development had proceeded to the antral stages. The treated mice regained normal, recurrent estrous cycles and continuously delivered pups for at least for 3 months, suggesting that LH-induced fibrosis in the ovarian stroma appears to alter the follicular-stroma microenvironment and restricts follicle growth at the secondary stage. Recently, Kawamura et al. [6] reported that the growth of secondary follicles to the multilayer stage in ovaries of low FSH-responding women was induced by the physical stimulation (cutting ovary) *in vivo*. They successfully promoted follicle growth and performed *in vitro* fertilization, and a healthy baby was delivered. The mechanisms by which mechanical stress appears to suppress follicle growth were explained by the YAP/TAZ mechano-transduction system in granulosa cells [7]. However, in the present study, the strong intensity of F-actin was only observed in the ovarian stroma and not within follicles. Therefore, further studies are required to determine the relationships among the abnormal endocrine condition, the accumulation of fibrotic tissue in ovarian stroma, and the suppression of follicular development in pre-antral follicles.

In conclusion, the appearance and maintenance of the aberrant endocrine cells and fibrous tissue within the ovarian stroma are dependent on chronic high levels of LH with increasing age. The accumulation of these cells and tissue in the ovarian stroma appears to impact surrounding secondary follicles leading to impaired follicular development by an FSH-independent manner. That long-term GnRH antagonist treatment normalized the endocrine functions and matrix conditions of the ovary and improved the fertility documents that the ovarian defects are LH-dependent. Thus, GnRH antagonist treatments might provide a new, noninvasive strategy for improving fertility in a subset of aging women before menopause.
